# Predicting susceptibility and resilience in an animal model of post-traumatic stress disorder (PTSD)

**DOI:** 10.1038/s41398-020-00929-9

**Published:** 2020-07-21

**Authors:** Paola Colucci, Enrico Marchetta, Giulia Federica Mancini, Phoebe Alva, Flavia Chiarotti, Mazahir T. Hasan, Patrizia Campolongo

**Affiliations:** 1 Department of Physiology and Pharmacology, Sapienza University of Rome, 00185 Rome, Italy; 2 Neurobiology of Behavior Laboratory, Santa Lucia Foundation, 00143 Rome, Italy; 3 Center for Behavioral Sciences and Mental Health, Istituto Superiore di Sanità, 00161 Rome, Italy; 4 Laboratory of Memory Circuits, Achucarro Basque Center for Neuroscience, 48940 Leioa, Spain; 5 Ikerbasque–Basque Foundation for Science, 48013 Bilbao, Spain

**Keywords:** Neuroscience, Psychiatric disorders

## Abstract

Post-traumatic stress disorder (PTSD) is a psychiatric disorder whose pathogenesis relies on a maladaptive expression of the memory for a life-threatening experience, characterized by over-consolidation, generalization, and impaired extinction, which are responsible of dramatic changes in arousal, mood, anxiety, and social behavior. Even if subjects experiencing a traumatic event during lifetime all show an acute response to the trauma, only a subset of them (susceptible) ultimately develops PTSD, meanwhile the others (resilient) fully recover after the first acute response. However, the dynamic relationships between the interacting brain circuits that might potentially link trauma-related experiences to the emergence of susceptible and resilient PTSD phenotypes in individuals is not well understood. Toward the first step to reach this goal, we have implemented our experimental PTSD model previously developed, making it suitable to differentiate between susceptible (high responders, HR) and resilient (low responders, LR) rats in terms of over-consolidation, impaired extinction, and social impairment long after trauma. Rats were exposed to five footshocks paired with social isolation. One week after trauma but before extinction, animals were tested in the Open Field and Social Interaction tasks for the identification of a predictive variable to identify susceptible and resilient animals before the possible appearance of a PTSD-like phenotype. Our findings show that exploratory activity after trauma in a novel environment is a very robust variable to predict susceptibility towards a PTSD-like phenotype. This experimental model is thus able to screen and differentiate, before extinction learning and potential therapeutic intervention, susceptible and resilient PTSD-like rats.

## Introduction

Post-traumatic stress disorder (PTSD) is a pathology with high prevalence and morbidity, the abnormal adaptation to a traumatic experience represents its specific pathogenic starting point^1–3^. The formation, consolidation, retrieval, and extinction of fear memories associated to a traumatic situation are orchestrated by multiple brain regions that act in a coordinate manner to elaborate the acute response to trauma^4–6^. After this normal acute response, most subjects fully recover. However, after experiencing a life-threatening event, in certain conditions the brain may switch to a maladaptive expression of memory specificities characterized by over-consolidation, memory generalization, and impaired extinction^7,8^. These cognitive alterations lead to a persistent reminding of the traumatic memories which in turn is responsible of PTSD development and consecutive symptoms (e.g., dramatic changes in arousal, mood, and social behavior)^1,3^. It has been estimated that more than 50% of the world population will encounter a trauma-causing experience once in their lifetime^9^ and even if all the traumatize persons will show an acute response to the trauma, the majority of them will recover without intervention^10^. Therefore, an important question arises on why some people do develop PTSD after a life-threatening experience whereas others do not.

It is well known that stress response cannot be sustained for a long time, the organism thus needs to develop effective physiological and psychological changes to cope with it^11^. Stressful experiences lead indeed to adaptation, but in some susceptible individuals this can be pathogenic^12,13^. The understanding of the neuronal mechanisms sustaining such pathogenic adaptation in susceptible subjects would hold the key for circuit-targeted therapeutics, especially due to the high rate of unresponsiveness or recrudescence following the initial remission of the pathology with the current treatments^14^.

Animal models of psychiatric diseases are useful tools for understanding the pathophysiological mechanisms of a specific disorder, thus contributing in the development of more effective therapeutic strategies for humans^15^. Although there are different rodent PTSD models, they however lack good translational value^16,17^. These animal models reproduce only the emotional symptoms of PTSD, such as hyperarousal and abnormal fear responses, thus neglecting the etiological causes of the pathology such as the cognitive alterations. However, a valid PTSD animal model should be able to simultaneously capture both the cognitive and emotional features of the pathology. Another important aspect is the chronicity issue of the pathology to sustain the behavioral alterations in an animal model long after trauma which has high relevance and translational value^18^. However, long-term changes are rarely investigated possibly because the trauma experience is not long-lasting. Finally, the interindividual variability in response to trauma is of outmost importance. Unfortunately, almost all PTSD animal models homogenize all the trauma-exposed animals as having the same phenotype, regardless of susceptibility or resilience to develop a PTSD-like phenotype, thus lacking construct and predictive validity^19^. In the spare studies that consider the individual variability to trauma, only the anxiety symptoms are used to discern between different PTSD-like phenotypes^20,21^, without considering the etiology (e.g. cognitive alterations such as the excessive memory consolidation and retrieval as well as the impaired extinction). Therefore, the development of an animal model which is able to predict individual differences in developing a chronic PTSD-like phenotype with regard of both cognitive and emotional alterations would be crucial to understand the neurobiological substrates underlying the individual variability towards PTSD development.

We developed an animal model that is able to capture both cognitive etiological alterations of PTSD together with a peculiar set of emotional dysfunctions related to the social domain, alterations resembling some of the core symptoms observed in the human pathology^22^. Here, we applied our animal model to identify susceptible or resilient individuals towards PTSD development; our model identifies a robust predictive variable to rigorously and systematically screen and differentiate, before extinction learning and potential therapeutic intervention, between susceptible (high responders, HR) and resilient (low responders, LR) rats in terms of over-consolidation, impaired extinction, and social impairment.

## Materials and methods

### Animal care and use

A total of 124 male adult Sprague-Dawley rats (350–450 g at the time of testing), Charles River Laboratories (Calco, Italy), were kept in an air-conditioned colony room (temperature: 21 ± 1 °C; lights on from 07:00 AM to 7:00 PM) with pellet food and water available ad libitum. Each rat was randomly assigned to the behavioral procedures. For every experiment, the exact sample size for each experimental group/condition is indicated in the figure legends. Sample size was carried out considering a power = 80%, the probability of committing a first type error (alpha) equal to 0.05 (G-power statistics). The experiments were performed in Sprague-Dawley rats because in the present study we adapted the PTSD animal model we first described, in which is used the strain of rat^22,23^. All the experiments were performed during the light phase of the cycle. Rats were handled for 1 min for three consecutive days before behavioral testing. All procedures involving animal care or treatments were performed in compliance with the ARRIVE guidelines, the Directive 2010/63/EU of the European Parliament, and the D. L. 26/2014 of Italian Ministry of Health.

### Behavioral procedures—Experiment 1

**Fig. 1:**
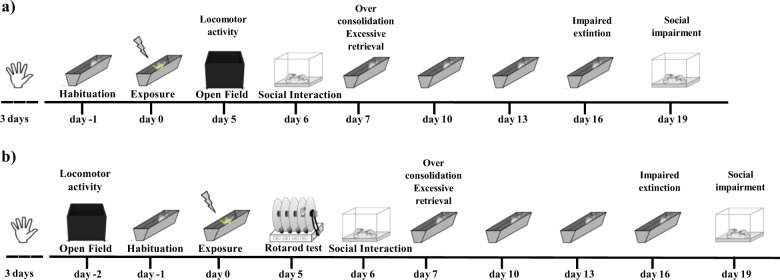
Experimental design. Behavioral procedures of Experiment 1 (**a**) and Experiment 2 (**b**).

The PTSD model we first described^22,23^ was adapted to make it suitable to distinguish between HR and LR animals in terms of over-consolidation, impaired extinction, and social impairment (for the experimental design see Fig. 1a). The experimental apparatus consisted in a metal trough shaped alley (60 cm long, 15 cm deep, 20 cm wide at the top, and 6.4 cm wide at the bottom) connected to an animal shocker. All the experimental sessions were video-recorded and subsequently scored by two well-trained researchers blind to the experimental conditions. After each session, fecal boli were removed and the apparatus was cleaned with a 70% ethanol solution.

#### Housing

All rats were individually housed for 2 days prior to the habituation session and remained singly housed until the end of the behavioral testing. We have previously shown that social isolation is necessarily required to develop enduring signs of emotional distress upon exposure to a traumatic event^22^.

#### Habituation

On the first day of testing (day −1), rats were individually taken from the home-cage and habituated for 5 min to the test apparatus. Rats were then returned to their home-cages.

#### Exposure session

On day 0, rats were individually placed in the apparatus for a total duration of 6 min, and were left undisturbed for the first 2 min. Then, 5 footshocks (2 s, 0.8 mA) were randomly delivered. After the last footshock rats were kept in the apparatus for 60 s to facilitate context association to the aversive stimuli.

#### Screening of HR and LR populations

Five and 6 days after trauma exposure rats were subjected to the Open Field and Social Interaction tests, respectively, with the purpose of identify a predictive variable for the screening of HR and LR phenotypes in accordance to their behavior.

#### Open Field test

Each rat was placed into the center of the Open Field arena made of black Plexiglas (80 × 80 × 60 cm) for 15 min. The test was performed under dim light conditions (2 lux). The total distance traveled (cm) by each rat, as well as the time spent in the center of the arena, were determined as parameters indicating the rat locomotor activity and emotionality, respectively. All the parameters here observed were acquired and analyzed using an automated video-tracking system (Smart, Panlab, Harvard Apparatus). After each session, rats were returned to their home-cage, fecal boli were removed, and the arena’s walls and floor were cleaned with a 70% ethanol solution.

#### Social Interaction test

In the social Interaction test couples of rats were put for 10 min in a quadrangular arena (40 × 40 × 60 cm) made of plexiglass with clean sawdust covering the floor, under red light conditions. After each session, rats were returned to their home-cage, fecal boli were removed, sawdust was blended, and the arena’s walls were cleaned with a 70% ethanol solution. Each couple of rats was established according to the unfamiliarity criteria (the two rats of each pair have never been housed in the same cage). In this test, social behavior was scored as previously described^22^.

#### Extinction sessions and extinction retention test

Each of the four extinction sessions consisted in a 10-min re-exposure to the experimental apparatus, with the first carried out 7 days after the exposure session (day 7). Each subsequent session was separated from the preceding one by a 72-h interval (days 10, 13, and 16). During each extinction session the contextual freezing behavior was evaluated^24^. Particularly, the freezing behavior analyzed during the first extinction session (7 days after trauma exposure) was considered as an index of the over-consolidation of memory related to the traumatic experience, whereas the last extinction session (16 days after trauma exposure) was considered as the extinction retention test, and the contextual freezing behavior here evaluated was considered as an index of the impaired extinction in our PTSD animal model.

#### Social Interaction test

To evaluate the social impairment, rats were tested in the Social Interaction test, which was carried out 72 h after the extinction retention test (day 19). Couples of rats for this second Social Interaction test were different from the previous couples in the Social Interaction test at 6 days after trauma exposure. The test was conducted as described above.

### Behavioral procedures—Experiment 2

In Experiment 2, a second group of rats was subjected to the rotarod test 5 days after trauma exposure (day 5), instead to the Open Field test, which was run 2 days before the trauma (day −2), accordingly with the experimental design shown in Fig. 1b. The Open Field test and the other behavioral procedures (habituation, exposure, extinction sessions, extinction retention test and Social Interaction test) as well as the Housing condition were run and maintained at the same way as described for Experiment 1.

#### Rotarod test

The Rotarod test was performed as previously described^25^. The rotarod speed was regularly accelerated each 30 s from 10 to 60 rpm. The cut-off time was fixed at 300 s. Rats were given three trials with 30 min inter-trial rest intervals. The mean time taken from each animal to fall from the rotarod apparatus, across the three trials, was measured and it represented a measure of the motor activity, since it is the time taken by each rat to maintain its balance walking on the revolving rod.

### Statistical analysis

Statistical analysis was performed using SPSS statistical software. Behavior was scored by three trained operators blinded to the animal phenotype and condition. Each measure is expressed as mean ± SEM. For each Pearson’s correlation performed, *R* coefficient and the relative *P* value were evaluated and a good correlation between variables was considered for *R* ≥ 0.35 and *P* < 0.05. Behavioral data were analyzed through a repeated measures ANOVA (RM ANOVA) or one-way ANOVA when appropriate. Tukey–Kramer post hoc test was performed to control for significant differences between groups and significance was considered for *P* < 0.05.

## Results

### Exploratory activity after trauma is a reliable predictive variable to identify resilient and susceptible animals towards a PTSD-like phenotype

**Fig. 2:**
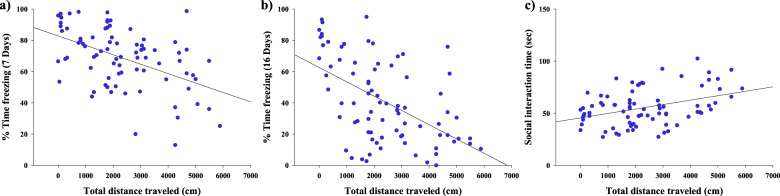
Exploratory activity after trauma is a robust predictive variable to identify resilient and susceptible animals towards a PTSD-like phenotype. The total distance traveled evaluated in the Open Field test performed 5 days after trauma exposure significantly correlated with freezing behaviors at 7 and 16 days after trauma, indexes of the over-consolidation, and impaired extinction of memory for the traumatic experience, respectively (**a**, **b**) and with the social interaction time, evaluated in the Social Interaction test performed 19 days after trauma, index of social/emotional alterations in the PTSD-like phenotype (**c**). *N* = 80.

In the first experiment, we aimed at identifying a predictive variable for HR and LR phenotypes screening. To this aim we performed a correlation analysis between potentially predictive variables (total distance traveled in the Open Field test, time spent in the center of the arena in the Open Field test, and time spent in social interaction in the Social Interaction test performed before the extinction learning) and other behavioral outcomes associated with PTSD (such as over-consolidation, excessive retrieval, impaired extinction, and social alterations). Particularly, we found a significant negative correlation between the total distance traveled, which is an index of the exploratory activity, in the Open Field test performed 5 days after trauma exposure and the freezing behavior shown by rats during the first extinction session (day 7) (*R* = −0.491, *P* < 0.0001) (Fig. 2a), index of the over-consolidation of the trauma experience in the PTSD model, and the freezing behavior shown by rats during the extinction retention test (day 16) (*R* = −0.532, *P* < 0.0001) (Fig. 2b), index of the impaired extinction of the trauma experience in the PTSD model. Thus, indicating that the less the rats explored the Open Field arena, the more they showed the freezing behavior during the first extinction session and the extinction retention test. Conversely, a significant positive correlation was found between the total distance traveled and the time spent in social interaction in the Social Interaction test performed 19 days after trauma exposure (*R* = 0.384, *P* < 0.001) (Fig. 2c) as an index of social alterations in the PTSD model. Thus, indicating that the less the rats explored the Open Field arena, the less they spent time in interacting with a conspecific during the Social interaction test. Subsequently, we performed the same correlation analysis, taking in consideration, as a potentially predictive variable, the time spent in the center of the Open Field arena during the Open Field test performed 5 days after trauma exposure, and no significant correlations were found between this parameter and the freezing behavior shown by rats during the first extinction session (day 7) (*R* = 0.011, *P* = 0.921, Supplementary Fig. 1a), the freezing behavior shown by rats during the extinction retention test (day 16) (*R* = 0.040, *P* = 0.727, Supplementary Fig. 1b) and the time spent in social interaction in the Social Interaction test performed 19 days after trauma exposure (*R* = 0.027, *P* = 0.812, Supplementary Fig. 1c). Finally, we performed the correlation analysis taking in consideration the time spent by each rat in social interaction, during the Social Interaction test performed 6 days after trauma exposure, as a potentially predictive variable. The statistical analysis revealed no significant correlations between this factor and the freezing behavior shown by rats during the first extinction session (day 7) (*R* = 0.107, *P* = 0.345, Supplementary Fig. 1d), the freezing behavior shown by rats during the extinction retention test (day 16) (*R* = 0.057, *P* = 0.617, Supplementary Fig. 1e), and the time spent in social interaction in the Social Interaction test performed 19 days after trauma exposure (*R* = 0.032, *P* = 0.776, Supplementary Fig. 1f).

For the statistical analysis of all the other parameters evaluated in the Open Field task, see Supplementary Results, Supplementary Figs. 2, 3, 4 and Supplementary Table 1.

### Rats classification according to their exploratory activity after trauma revealed behavioral alterations associated with the PTSD-like phenotype

**Fig. 3:**
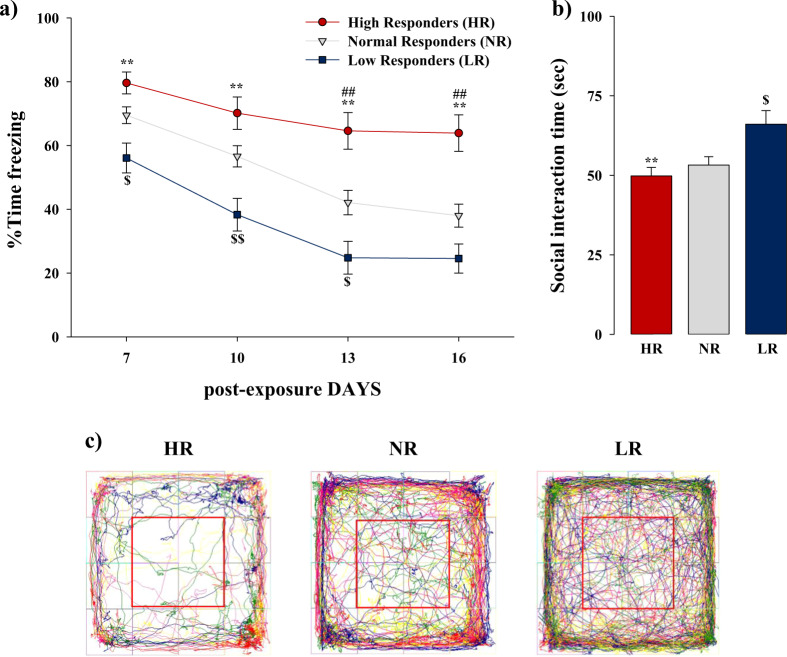
Rats classification according to their exploratory activity after trauma revealed behavioral alterations associated with the PTSD-like phenotype in rats. Freezing rates across the three extinction sessions (days: 7, 10, and 13) and the extinction retention test (day 16) in rats segregated in HR (susceptible), NR (normal), and LR (resilient) according to the 75th and 25th percentile of the experimental group’s distribution for the total distance traveled in the Open Field test performed 5 days after trauma exposure. HR rats displayed increased freezing response compared to LR rats, across all the extinction sessions and still at extinction retention test, where they showed higher level of freezing also with respect to the NR group. LR rats showed an exactly opposite freezing profile, with lower levels of freezing across all the extinction session and no significant differences with respect to the NR group at the extinction retention test. **a** Social interaction time of HR, NR, and LR rats. HR and NR rats spent less time interacting with a conspecific with respect to LR rats. **b** Representative path tracks of five animals per group indicating the exploratory activity in the Open Field arena of HR, NR, and LR rats (**c**). ^§^*P* < 0.05 LR vs NR group; **P < 0.01 HR vs LR group; ^##^*P* < 0.01 HR vs NR group; ^§§^*P* < 0.01 LR vs NR group. *N* = 80 (20 HR, 40 NR, 20 LR).

Thereafter the identification of the total distance traveled in the Open Field test as a promising predictive variable for the HR and LR phenotypes screening, we defined the rats’ classification according to the extremes (25th or 75th percentile) of the experimental group’s distribution for this parameter. Each rat scored above 25th or over 75th percentiles has been considered as susceptible (HR) or resilient (LR), respectively, whereas each rat scored between 25th and 75th percentiles has been considered normal responder (NR). The subsequent RM ANOVA for the freezing behavior analysis across the extinction sessions in the three experimental groups (Fig. 3a) revealed a significant effect of the phenotype HR, LR, or NR (*F*_(2,77)_ = 18.189, *P* < 0.0001), a significant effect of the extinction sessions (*F*_(3,77)_ = 48.510, *P* < 0.0001) and tendency toward significance for the interaction between these two factors (*F*_(6,231)_ = 2.071, *P* = 0.058). Post hoc tests showed that HR rats presented higher levels of freezing across the extinction sessions and at extinction retention test as compared with the LR group (*P*s < 0.01, for all days of extinction), and they presented higher levels of freezing with respect to the NR group only during the third extinction session (day 13) and the extinction retention test (day 16) (*P*s < 0.01). Moreover, the post hoc tests revealed that the LR group presented lower levels of freezing across all the extinction sessions as compared with the NR group (*P* < 0.05, days 7 and 13; *P* < 0.01, day 10), excepted for the extinction retention test (day 16) in which no significant differences between these two groups were found. Then, through a one-way ANOVA analysis, we analyzed the time spent in social interaction during the Social Interaction test performed 19 days after the trauma exposure (Fig. 3b) and a significant difference between the three experimental groups has been revealed (*F*_(2,77)_ = 5.801, *P* = 0.005). Particularly, post hoc analysis revealed that HR and NR groups spent less time in social interaction with respect to the LR group (*P* < 0.01 and *P* < 0.05, respectively). The representative path tracks indicating the exploratory activity in the Open Field arena of HR, NR, and LR rats are shown in Fig. 3c.

### Trauma induced changes in the natural tendency to explore a new environment is a specific variable for the screening of a PTSD-like phenotype

In the second experiment, we aimed at evaluating whether is the pure motor activity to predict a PTSD-like phenotype rather than the trauma induced changes to exploratory activity. To this aim, we performed a correlation analysis between the mean time taken by each animal to fall from the rotarod apparatus (a pure measure of motor activity) and the behavioral outcomes associated with PTSD. The same correlation analysis was also performed between the total distance traveled in the Open Field test before trauma and the behavioral outcomes associated with the PTSD-like phenotype. The correlation analysis between the mean time across the three trials, for each animal to fall from the rotarod apparatus and the freezing behavior shown by rats during the first extinction session (day 7, Supplementary Fig. 5a) and the extinction retention test (day 16, Supplementary Fig. 5b) did not reveal any significant correlation (*R* = 0.133, *P* = 0.389; *R* = 0.106, *P* = 0.494; respectively). No significant correlation was also found between this parameter of pure motor activity and the time spent in social interaction during the Social Interaction test performed 19 days after trauma exposure (*R* = 0.073, *P* = 0.637, Supplementary Fig. 5c). The correlation analysis between the total distance traveled in the Open Field arena before trauma and the behavioral outcomes associated with the PTSD-like phenotype did not revealed any significant correlation between this parameter and the freezing behavior shown during the first extinction session (day 7) (*R* = 0.047, *P* = 0.760, Supplementary Fig. 5d), the freezing behavior shown during the extinction retention test (day 16) (*R* = 0.036, *P* = 0.817, Supplementary Fig. 5e) and the time spent in social interaction during the Social Interaction test performed 19 days after trauma (*R* = 0.081, *P* = 0.600, Supplementary Fig. 5f).

For the statistical analysis of all the other parameters evaluated in the Open Field task see Supplementary Results and Supplementary Table 2.

## Discussion

In the present study, by using specific behavioral tests suitable to map the individual differences in an experimental model of PTSD, we found a robust predictive variable for the screening of PTSD susceptibility immediately after trauma and before therapeutic intervention. We found the level of exploratory activity soon after trauma in a novel environment to be a robust and reliable predictive variable to identify later susceptibility towards PTSD development. The analysis of the locomotor activity in a novel arena is an easy and valuable parameter to simultaneously evaluate both motor activity and the natural tendency of rodents to explore a new environment^26^. Through a correlation analysis, we found a negative dependence between this parameter and all the memory alterations clusters in animals presenting a PTSD-like phenotype: i.e. over-consolidation and impaired extinction (represented by the percentage of time spent in freezing behavior, during the first extinction session 1 week after trauma, and after the last extinction session 16 days after trauma). Maladaptive memory processes are specific traits of PTSD pathology^2^ which can be easily reproduced in rodents to investigate their neuronal underpinnings^22^. Contextual freezing behavior represents a fear and anxiety related response shown by rodents when exposed to the context in which they experienced trauma^27^. This behavior is linked to fear memory retention. The fear memory elicited during the first extinction session is a measure of memory retention and its retrieval. In fact, at this time point, the extinction learning has not started yet, and an excessive memory retrieval can be due to a maladaptive memory consolidation processes induced by the traumatic experience and known as over-consolidation^28^. Conversely, freezing behavior during the latter extinction retention test represents an index of deficit in fear extinction of the traumatic experience^29^. Our results highlight that the lower is the level of exploratory activity after trauma the higher is the freezing behavior of rats both at the retrieval or extinction sessions (indicative of exaggerated retention and lack of extinction). This result highlights this behavioral parameter as strictly related to the PTSD-like cognitive maladaptive changes of over-consolidation and impaired extinction.

One of the overarching features of PTSD in humans, in addition to the cognitive alterations previously mentioned, is social impairment^30^. Several clinical reports indicate social withdrawal and social isolation as common hallmarks of PTSD patients^31–33^. In the animal model that we had previously validated, we found that rats exposed to footshock trauma paired with social isolation displayed reduced social interaction time in the Social Interaction test and impaired memory processes^22^. Here, we found that the exploratory activity of a novel environment soon after trauma not only correlates with cognitive alterations but it also positively correlates with the time spent in social interaction long after trauma exposure. The lower is the exploratory activity the lower is the level of social interaction, thus making this parameter a predictive variable also for the trauma induced maladaptive changes in the emotional domains long after stress exposure. Taken together these results indicate the level of exploratory activity used as a very robust predictive variable to immediately identify subjects that will develop chronic PTSD-like alterations later after trauma.

We thus hypothesize that trauma experience enhances the stress responsiveness in susceptible rats to other potentially stressful situation, such as a novel environment and, as a consequence of this, a reduction of their innate tendency for exploration as a form of fear generalization. In support of this hypothesis, we found a different habituation profile in the total distance traveled in the Open Field arena across time among the groups tested before or after trauma. While rats tested before trauma showed a normal habituation profile to the arena with higher explorative activity during the first phase of the test, traumatized rats did not. Importantly, susceptible rats explored less the Open Field arena for all the test duration, whereas the NRs and resilient ones moved less at the beginning but started to explore more the arena towards the end of the test displaying an opposite habituation profile with respect to rats not exposed to trauma. This phenotype-specific habituation profile has a high translational value and may be ascribable to fear generalization induced by the traumatic experience, which are key features of PTSD and which consist in the transfer of fear to stimuli not related to the aversive event^34–37^.

To further test the robustness of the identified predictive variable, we segregated animals in susceptible, normal and resilient, according to the 25th and 75th percentile of the experimental group’s distribution for the exploratory activity in the Open Field test. Our results indicate that susceptible rats display increased freezing response compared to resilient rats, across retrieval and extinction sessions, where they show higher level of freezing also with respect to the group exerting normal levels of activity. Thus, resembling the PTSD human clusters, where an over-consolidation of fear memory of the trauma and an impaired extinction learning after exposure therapy sessions are robust endophenotypes of susceptibility to PTSD pathology, whereas, the absence of these maladaptive changes is typical of a resilient phenotype^38^. The same classification revealed significant differences among experimental groups also in the Social Interaction test. Particularly, we found that susceptible rats spent less time interacting with a conspecific after trauma with respect to the resilient ones. These findings add a high translational value to our PTSD animal model with respect to human PTSD.

Motor activity has a good translational value since it may resemble the strategies adopted by human subjects to deal with stressful situations, known as coping strategies^39–41^. For a rat the distance traveled in a new arena, as previously mentioned, is a measure of not only motor activity but also of the innate tendency of the animal to explore a new environment moving across it^26^. Literature data indicate a reduction in the motor activity in animals exposed to contextual fear conditioning regardless of their individual susceptibility^42,43^. Prompted by this evidence and results, that we obtained in the first set of experiments, we next aimed to dissociate these two aspects, in order to better understand if it is the motor activity per se, or the tendency to explore a new environment that made the exploratory behavior after trauma a reliable predictive variable for the development of a PTSD-like phenotype. We thus analyzed pure motor behavior in the rotarod test, but we did not find any significant correlation between the pure motor activity assessed in this test and the behavioral alterations associated with the PTSD-like phenotype, thus excluding the role of the pure motor activity in making the exploratory behavior soon after trauma a predictive variable for the development of a PTSD-like phenotype.

We then aimed at clarifying whether the natural tendency to explore an environment regardless of any trauma exposure would be useful to disentangle between resilient and susceptible animals or is it rather the reaction after trauma that, by soon influencing the exploratory behavior, becomes a strong predictive variable to screen between the two phenotypes before the pathology develops. Interestingly, we did not find any significant correlation between the exploratory activity in the Open Field test performed before the traumatic experience and any behavioral outcome associated with the PTSD-like phenotype. These results clearly indicate that only the change induced by the trauma exposure on the exploratory behavior before the pathology occurs make it a predictive variable to soon identify animals susceptible to later develop a PTSD-like phenotype.

The Anxiety and Depression Association of America (ADAA)^44^ has estimated that physical exercise (e.g. running and walking) and talking to friends are among the most common ways people adopt to cope with stress after trauma. Interestingly, literature data indicate that people with PTSD participate less to physical activity^45–47^. Not only people with PTSD are less physical active, but with respect to people who fully recover after trauma, they also tend to stay at home for longer time and are less interested in exploring new places and in living new experiences, because any kind of stimuli in an unfamiliar environment can trigger suppressed or unwanted memories and emotions^48^. In consideration of the translational value of our present results, it is a tentative to speculate that it could be theoretically possible to detect resilient and susceptible humans’ individuals in later developing PTSD, on the basis of their attitude after trauma to soon cope with stress by practicing physical activity and by their willingness to search for new experiences in unfamiliar environments^45–47^.

In conclusion, we here developed a rat animal model able to predict individual differences in later developing a PTSD-like phenotype with a high translational value with respect to the cognitive and emotional clusters observed in the human pathology. Overall, our results pave the road for further studies in which susceptible and resilient animals can be differentially manipulated at the interacting circuit-levels to a better understand the neurobiological underpinnings of susceptibility and resilience towards PTSD development, providing pre-clinical prospects with therapeutic interventions in a rat animal model and, subsequently, translated to humans.

## Supplementary information

Supplementary Results

Supplementary Fig. 1

Supplementary Fig. 2

Supplementary Fig. 3

Supplementary Fig. 4

Supplementary Fig. 5

Supplementary Tables
