# Supplementary material for: Predicting susceptibility and resilience in an animal model of post-traumatic stress disorder (PTSD)
Source: Transl Psychiatry. 2020 Jul 21;10:243. doi: 10.1038/s41398-020-00929-9 (PMC7374603; doi:10.1038/s41398-020-00929-9)
Supplement: Supplementary file 1 — Supplementary Results [file 41398_2020_929_MOESM1_ESM.docx]

**Supplementary Results**

Data of Supplementary Fig. 1 and 5 were discussed in the main text.

**Habituation profile of total distance traveled in the Open Field arena**

To analyze differences in the intrasession habituation in the Open Field task, we evaluated the distance traveled across three blocks of 5 min time sequences each in HR, NR and LR rats tested in the Open Field after trauma and in a group of rats tested in the Open Field before trauma. Overall, these results suggested the existence of a different habituation profile among the experimental groups, which was influenced by the trauma-induced phenotype. While rats tested before trauma showed a normal habituation profile to the arena with higher explorative activity during the first phase of the test, traumatized rats did not. It is interesting to note that HR rats explored less the Open Field arena for all the three intrasession intervals, whereas the NR and LR groups moved less at the beginning of the test but started to explore the arena towards the end of the test displaying an opposite habituation profile with respect to rats not exposed to trauma.

RM ANOVA for the intrasession habituation in the Open Field arena (expressed as distance traveled in three blocks of 5 min time sequences) (Supplementary Fig. 2a), revealed significant effects of the experimental group (F_(3, 120)_ = 153.024, P < 0.0001), distance traveled (F_(2, 120)_ = 5.121, P = 0.007) and interaction between these two factors (F_(6, 240)_ = 34.944, P < 0.0001). Post hoc tests showed that in the first block, HR rats presented lower distance traveled compared to LR rats (P < 0.01), NR rats (P < 0.05) and rats tested before trauma (P < 0.01). Moreover, LR rats showed higher distance traveled than NR rats (P < 0.01), and both LR and NR rats presented lower distance traveled with respect to rats tested before trauma (P < 0.01). Post hoc tests showed that in the second block, HR rats presented lower distance traveled compared to LR rats (P < 0.01), NR rats (P < 0.01) and rats tested before trauma (P < 0.01). In addition, LR rats showed higher distance traveled than NR rats (P < 0.01), and NR rats presented lower distance traveled respect to rats tested before trauma (P < 0.01). Post hoc tests showed that in the third block, HR rats presented lower distance traveled compared to LR rats (P < 0.01), NR rats (P < 0.01) and rats tested before trauma (P < 0.01). Moreover, LR rats showed higher distance traveled than NR rats (P < 0.01) and rats tested before trauma (P < 0.01). We also evaluated the total distance traveled in the Open Field task in HR, NR and LR rats tested in the Open Field after trauma and in rats tested in the Open Field before trauma. One-way ANOVA for the total distance traveled among these experimental groups (Supplementary Fig. 2b), revealed a significant statistical difference (F_(3, 120)_ = 153.024, P < 0.0001). Post hoc tests indicated that HR rats presented lower total distance traveled compared to LR rats (P < 0.01), NR rats (P < 0.01) and rats tested before trauma (P < 0.01). Further, LR rats presented higher total distance traveled with respect to NR rats (P < 0.01) and NR rats showed lower total distance traveled than the group of rats tested before trauma (P < 0.01).

**Exploratory activity after trauma is a reliable predictive variable to identify resilient and susceptible animals towards a PTSD-like phenotype**

In the first experiment we found that total distance traveled in the Open Field task after trauma is a reliable predictive variable to identify HR and LR animals towards a PTSD-like phenotype. Therefore, we performed a more detailed statistical analysis by using correlation analyses between distance traveled across the three blocks of 5 min time sequences in the Open Field task after trauma and the behavioral outcomes associated with PTSD: over-consolidation, impaired extinction and social alterations, expressed by freezing behavior during the extinction sessions at 7 and 16 days after trauma exposure in the PTSD model and sociability in the Social Interaction test performed 19 days after trauma. Our results indicated significant negative correlations between distance traveled during the first block of 5 min time in the Open Field task performed 5 days after trauma and freezing behavior shown by rats during the first extinction session (day 7) (R = - 0.356, P = 0.001) (Supplementary Fig. 3a) and during the extinction retention test (day 16) (R = - 0.404, P < 0.001) (Supplementary Fig. 3b). Conversely, a significant positive correlation was found between distance traveled in the first block of 5 min time in the Open Field task performed 5 days after trauma and the time spent in social interactions 19 days after trauma exposure (R = 0.288, P = 0.010) (Supplementary Fig. 3c). In line with these results, we found significant negative correlations between distance traveled during the second block of 5 min time in the Open Field task performed 5 days after trauma exposure and freezing behavior shown by rats during the first extinction session (day 7) (R = - 0.407, P < 0.001) (Supplementary Fig. 3d) and during the extinction retention test (day 16) (R = - 0.434, P < 0.0001) (Supplementary Fig. 3e). Conversely, a significant positive correlation was found between distance traveled in the second block of 5 min time in the Open Field task performed 5 days after trauma and the time spent in social interactions 19 days after trauma exposure (R = 0.366, P < 0.001) (Supplementary Fig. 3f). We also found significant negative correlations between distance traveled during the third block of 5 min time in the Open Field task performed 5 days after trauma and freezing behavior shown by rats during the first extinction session (day 7) (R = - 0.411, P < 0.001) (Supplementary Fig. 3g) and during the extinction retention test (day 16) (R = - 0.441, P < 0.0001) (Supplementary Fig. 3h). Conversely, a significant positive correlation was found between distance traveled in the third block of 5 min time in the Open Field task performed 5 days after trauma and the time spent in social interactions 19 days after trauma exposure (R = 0.267, P = 0.017) (Supplementary Fig. 3i). These results as a whole indicate that the less rats explored the Open Field arena independently of the 3 blocks of 5 min time sequences, the more they showed freezing behavior during extinction, and that the less the rats explored the Open Field arena independently of the three blocks of 5 min time sequences, the less they spent time in interacting with a conspecific during the Social interaction test.

In order to demonstrate that total distance traveled is a predictive variable for HR and LR phenotypes screening, in addition to the statistical analysis already presented in the main manuscript, we performed a correlation analysis between other potentially predictive variables evaluated in the Open Field task and the behavioral outcomes associated with PTSD (such as: over-consolidation, excessive retrieval, impaired extinction and social alterations). The results obtained indicated significant correlations between the time spent in vertical locomotor activity (rearing activity) and freezing behavior shown by rats during the first extinction session (day 7) (R = - 0.465, P < 0.0001) (Supplementary Fig. 4a) index of the over-consolidation of the trauma experience, the freezing behavior shown by rats during the extinction retention test (day 16) (R = - 0.447, P < 0.0001) (Supplementary Fig. 4b), index of the impaired extinction of the trauma and the time spent in social interaction 19 days after trauma exposure (R = 0.246, P = 0.03) (Supplementary Fig. 4c) as an index of social and emotional alterations. The same results were obtained for the correlation analysis between the frequency of rearing and freezing behavior shown by rats during the first extinction session (day 7) (R = 0.508, P < 0.0001) (Supplementary Fig. 4d), during the extinction retention test (day 16) (R = 0.501, P < 0.0001) (Supplementary Fig. 4e) and the time spent in social interaction 19 days after trauma exposure (R = 0.320, P = 0.004) (Supplementary Fig. 4f). Finally, the results obtained indicated statistical significant correlations between the time spent in immobility and freezing behavior shown by rats during the first extinction session (day 7) (R = 0.427, P < 0.0001) (Supplementary Fig. 4g), the freezing behavior shown by rats during the extinction retention test (day 16) (R = 0.562, P < 0.0001) (Supplementary Fig. 4h), and time spent in social interaction 19 days after trauma exposure (R = - 0.284, P = 0.01) (Supplementary Fig. 4i). The non-statistically significant results obtained from the analysis of all the other behavioral parameters evaluated in the Open Field task are listed in Supplementary Table 1.

**Trauma induced changes in the natural tendency to explore a new environment is a specific variable for the screening of a PTSD-like phenotype**

In the second experiment, we aimed at evaluating whether is the pure motor activity to predict a PTSD-like phenotype rather than the trauma induced changes to exploratory activity. The correlation analysis between all the parameters evaluated in the Open Field test performed before the trauma exposure, such as the vertical locomotor activity evaluated through the rearing activity (frequency and time), grooming (frequency and time), immobility (time) and fecal boli deposition, and the behavioral outcomes associated with PTSD (such as: over-consolidation, excessive retrieval, impaired extinction and social alterations) did not reveal any statistical significant correlation. The results obtained are summarized in Supplementary Table 2.

**Supplementary Figure legends**

**Supplementary Figure 1. Correlation analysis considering the time spent in the center of the Open Field arena and the Social Interaction Time as predictive variables in term of resilience and susceptibility to develop a PTSD-like phenotype after trauma exposure in rats.** No significant correlation was found between the time spent in the center of the Open Field arena and the freezing behavior at 7 and 16 days after trauma, index of the over-consolidation and impaired extinction of memory for the traumatic experience, respectively **(a, b)** and with the social interaction time evaluated in the Social Interaction test performed 19 days after trauma exposure as an index of social behavior alterations in the PTSD-like phenotype **(c)**. No significant correlation has been revealed between the time spent in social interaction in the Social Interaction test performed 6 days after trauma and the freezing behavior at 7 and 16 days after trauma, index of the over-consolidation and impaired extinction of memory for the traumatic experience, respectively **(d, e)** and with the social interaction time evaluated in the Social Interaction test performed 19 days after trauma exposure as an index of social behavior alterations in the PTSD-like phenotype **(f)**. N = 80.

**Supplementary Figure 2. Habituation profile of total distance traveled in the Open Field arena.** Distance traveled across three blocks of 5 min time sequences in HR, NR and LR rats tested in the Open Field task 5 days after trauma and in rats tested in the Open Field task before trauma **(a)**. Total distance traveled in HR, NR and LR rats tested in the Open Field after trauma and in rats tested in the Open Field task before trauma **(b)**. #, P < 0.05 and ##, P < 0.01 HR vs NR group; §§, P < 0.01 LR vs NR group; **, P < 0.01 HR vs LR group; ++, P < 0.01 HR vs pre-trauma group; °°, P < 0.01 LR vs pre-trauma group; çç, P < 0.01 NR vs pre-trauma group. N = 44-80 per group.

**Supplementary Figure 3. Exploratory activity after trauma is a reliable predictive variable to identify resilient and susceptible animals towards a PTSD-like phenotype.** Correlation analyses between distance traveled across the three blocks of 5 min time sequences in the Open Field performed 5 days after trauma and the freezing behavior at 7 (**a, d, g**) and 16 days (**b, e, h**) after trauma in the PTSD model, and social interaction time 19 days after trauma (**c, f, i**). N = 80.

**Supplementary Figure 4. Correlation analysis of rearing activity and immobility with PTSD-like alterations.** The time spent in rearing activity in the Open Field arena performed 5 days after trauma significantly correlated with freezing behaviors at 7 and 16 days after trauma, indexes of the over-consolidation and impaired extinction of memory for the traumatic experience, respectively **(a, b)** and with the social interaction time, evaluated in the Social Interaction test 19 days after trauma, index of social/emotional alterations in the PTSD-like phenotype **(c).** The frequency of rearing activity in the Open Field arena performed 5 days after trauma significantly correlated with freezing behaviors at 7 and 16 days after trauma, respectively **(d, e)** and with the social interaction time, evaluated in the Social Interaction test 19 days after trauma **(f)**. The time spent in immobility in the Open Field arena performed 5 days after trauma significantly correlated with freezing behaviors at 7 and 16 days after trauma, respectively **(g, h)** and with the social interaction time, evaluated in the Social Interaction test 19 days after trauma **(i).** N = 80.

**Supplementary Figure 5. Correlation analysis considering the time to fall from the rotarod apparatus and the total distance traveled in the Open Field test performed before trauma exposure.** No significant correlation has been identified between the mean time to fall from the rotarod apparatus and the freezing behavior at 7 and 16 days after trauma, index of the over-consolidation and impaired extinction of memory for the traumatic experience, respectively **(a, b)** and with the social interaction time evaluated in the Social Interaction test performed 19 days after trauma exposure as an index of social behavior alterations in the PTSD-like phenotype **(c)**. No significant correlation was found between the time spent in center of the Open Field arena in the Open Field test performed 2 days before trauma and the freezing behavior at 7 and 16 days after trauma, index of the over-consolidation and impaired extinction of memory for the traumatic experience, respectively **(d, e)** and with the social interaction time evaluated in the Social Interaction test performed 19 days after trauma exposure as an index of social behavior alterations in the PTSD-like phenotype **(f)**. N = 44.
