# Supplementary figures and images for: Predicting susceptibility and resilience in an animal model of post-traumatic stress disorder (PTSD)
Source: Transl Psychiatry. 2020 Jul 21;10:243. doi: 10.1038/s41398-020-00929-9 (PMC7374603; doi:10.1038/s41398-020-00929-9)

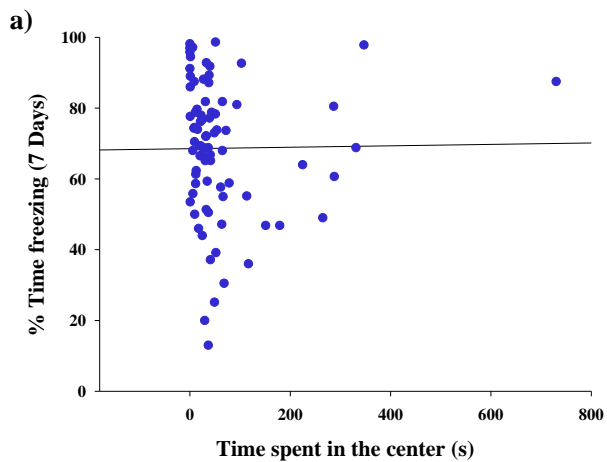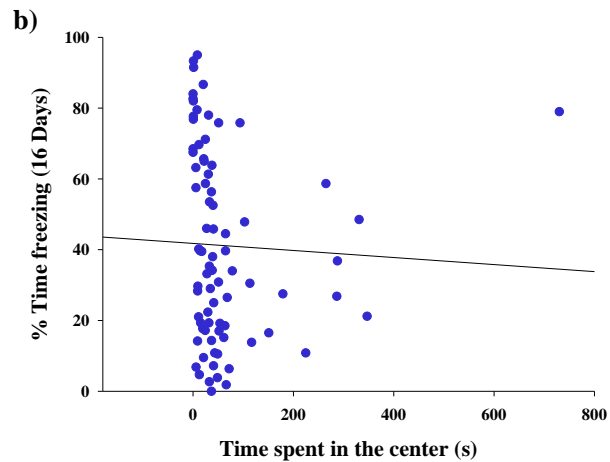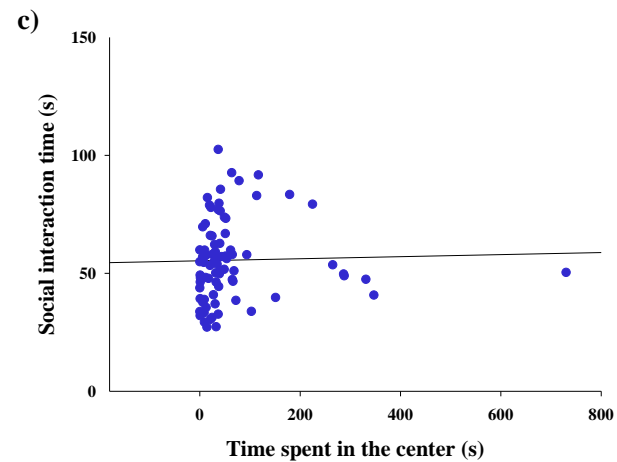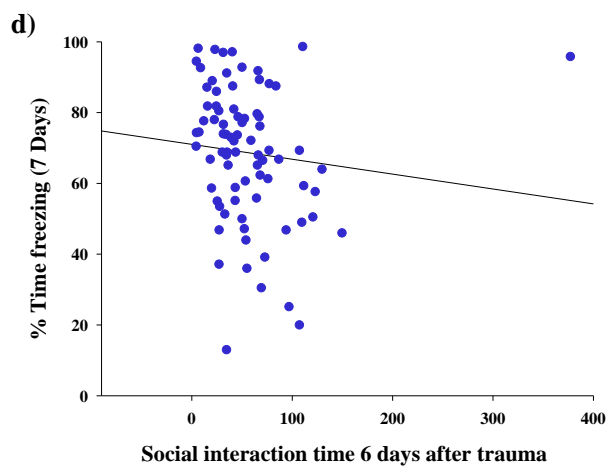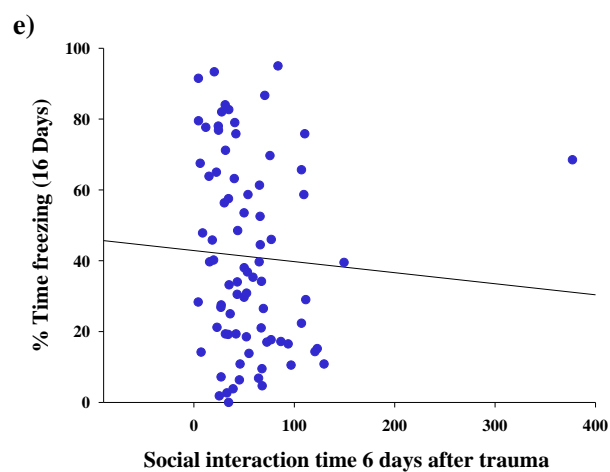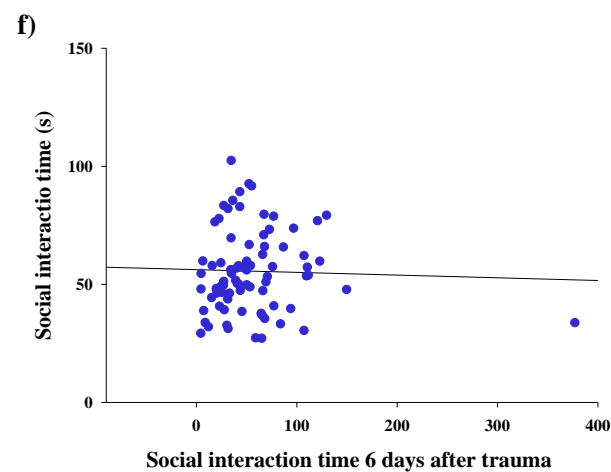

Supplement: Supplementary file 2 — Supplementary Fig. 1 [file 41398_2020_929_MOESM2_ESM.pdf]

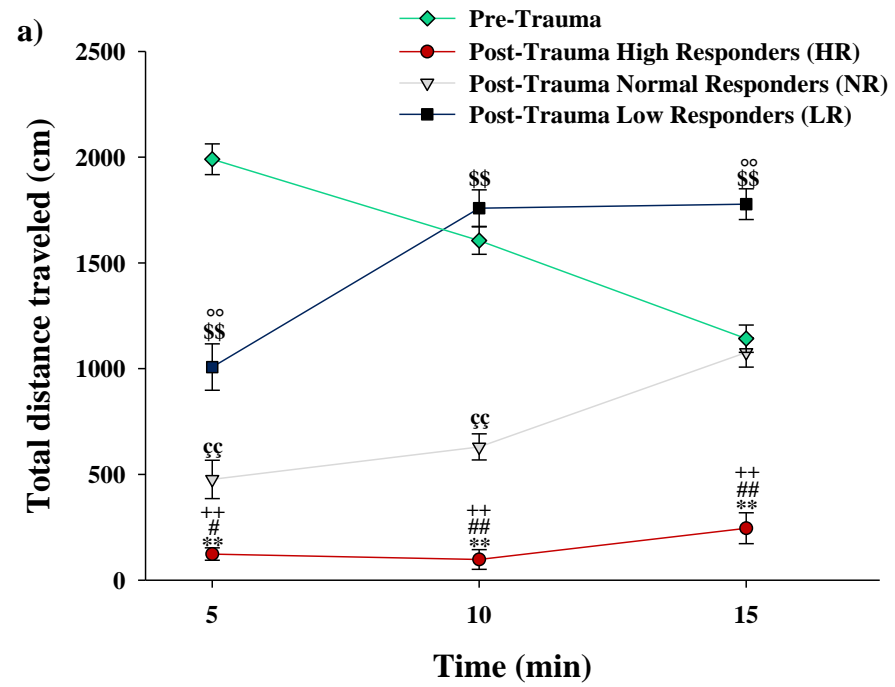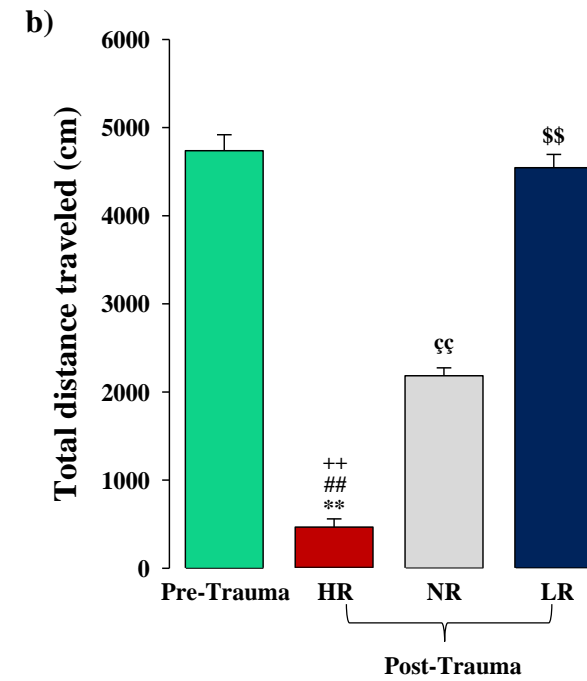

Supplement: Supplementary file 3 — Supplementary Fig. 2 [file 41398_2020_929_MOESM3_ESM.pdf]

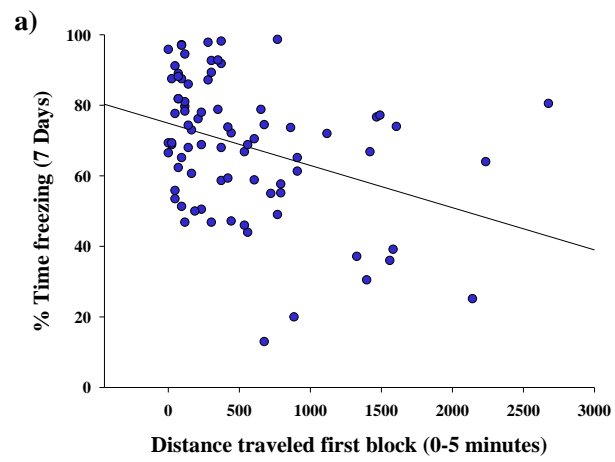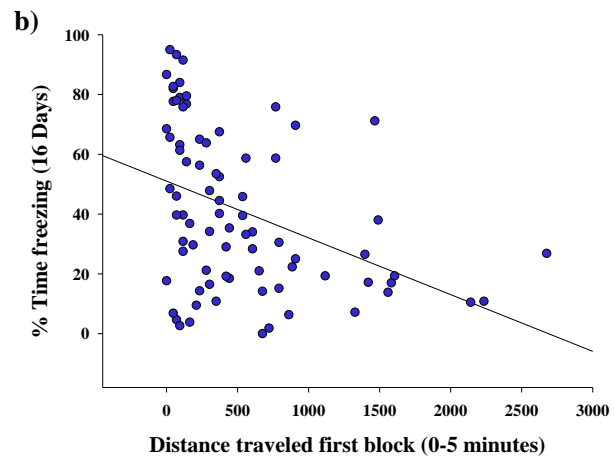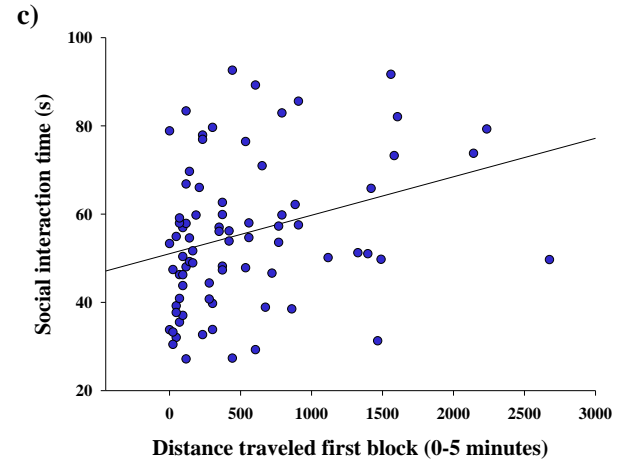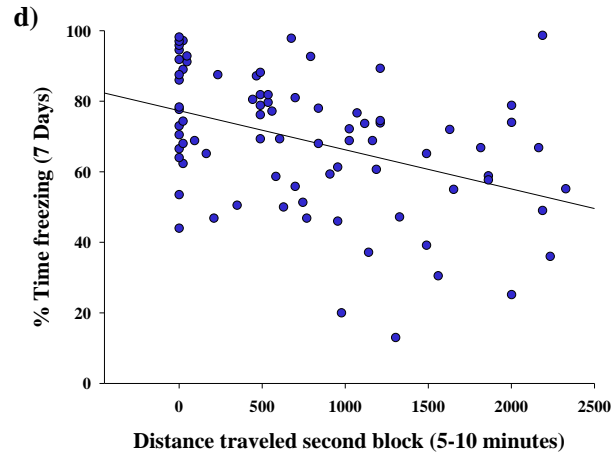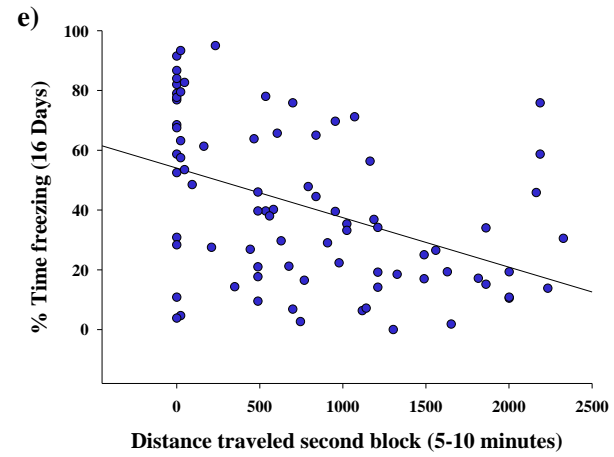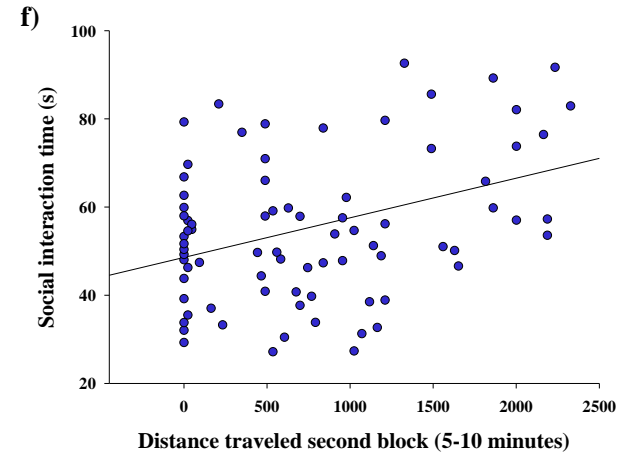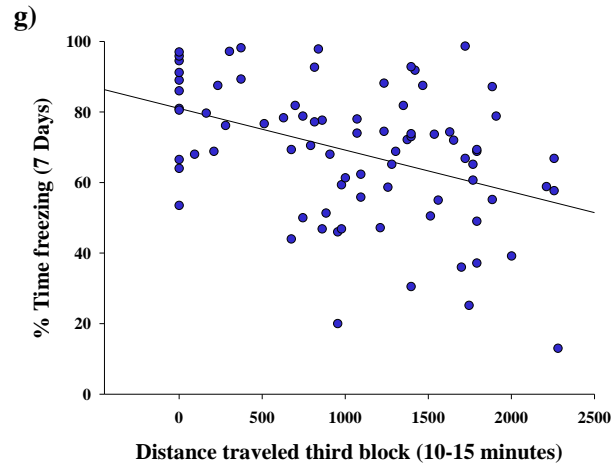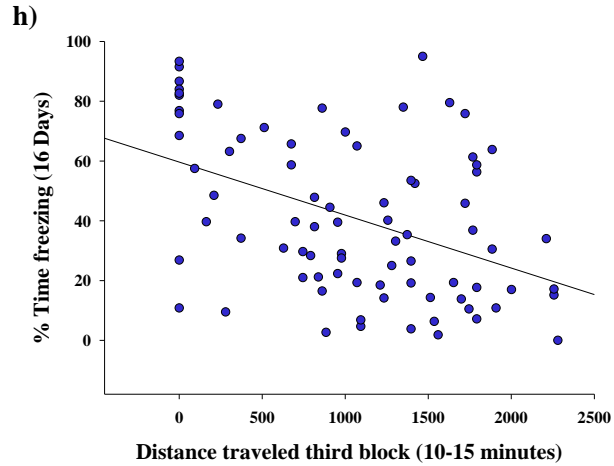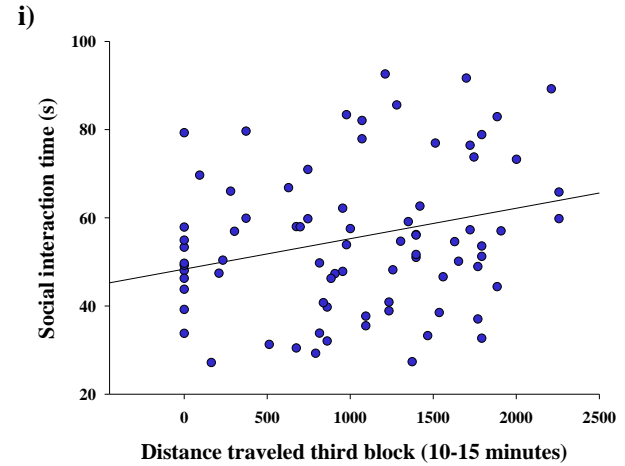

Supplement: Supplementary file 4 — Supplementary Fig. 3 [file 41398_2020_929_MOESM4_ESM.pdf]

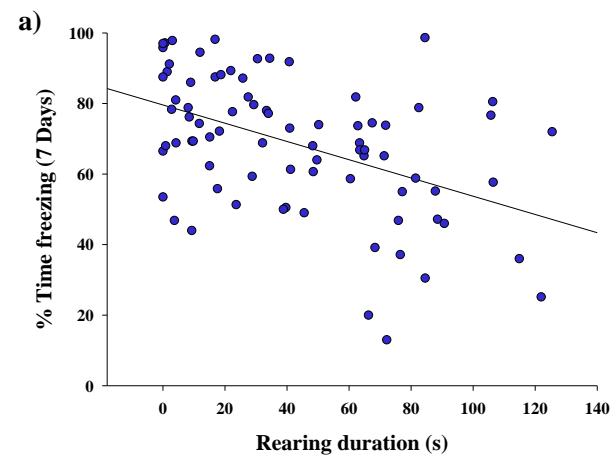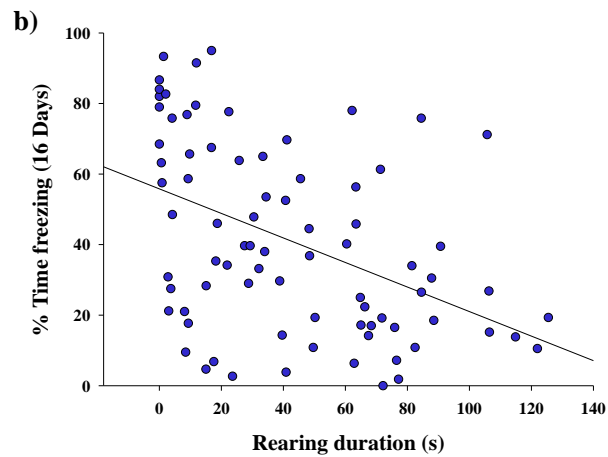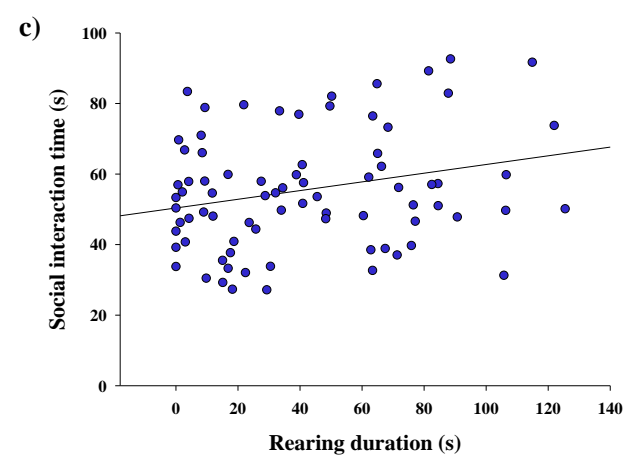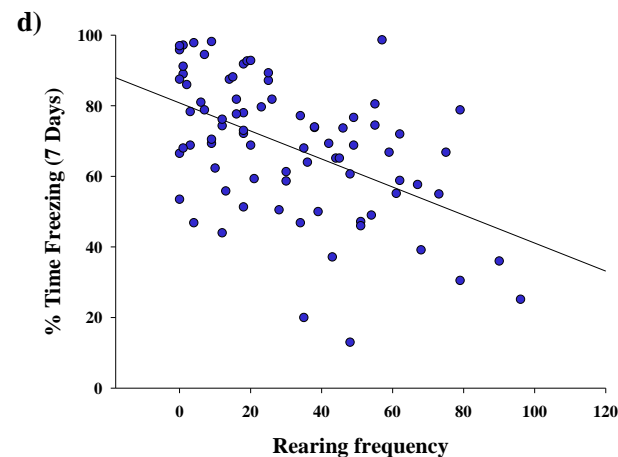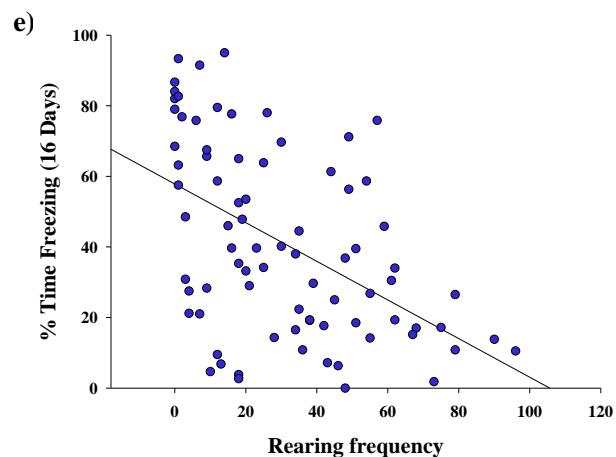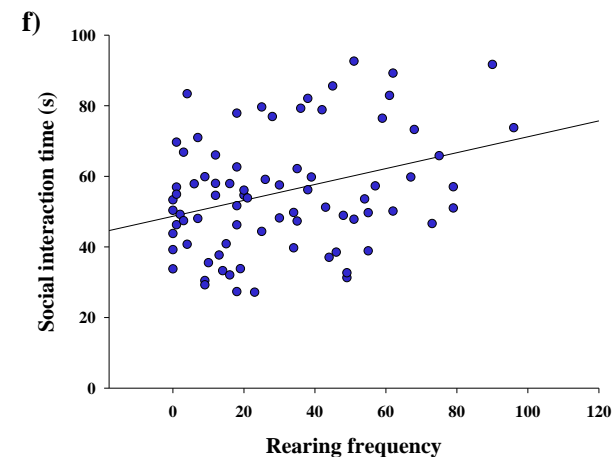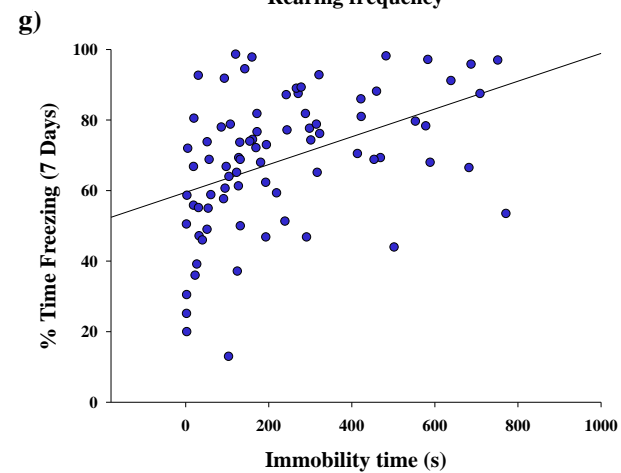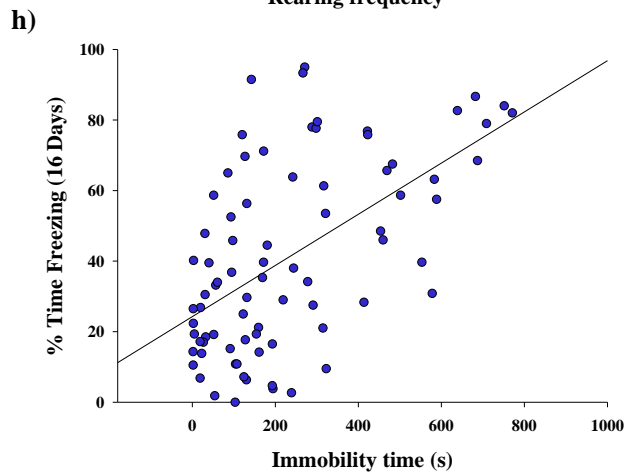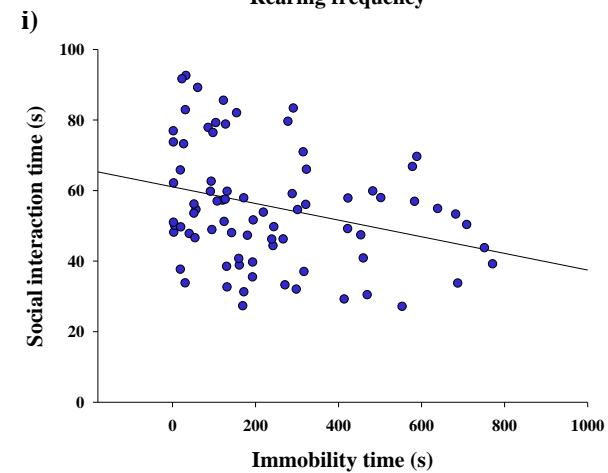

Supplement: Supplementary file 5 — Supplementary Fig. 4 [file 41398_2020_929_MOESM5_ESM.pdf]

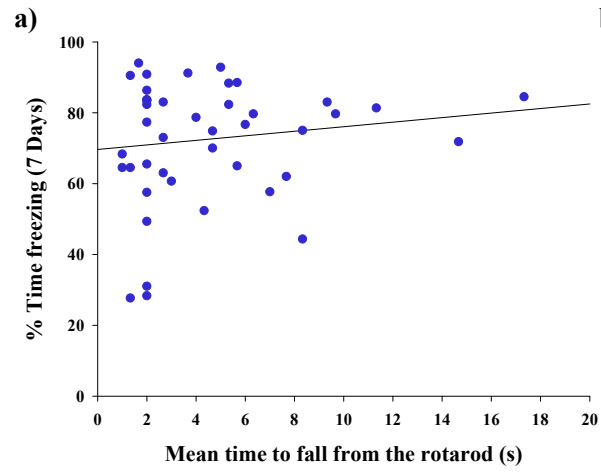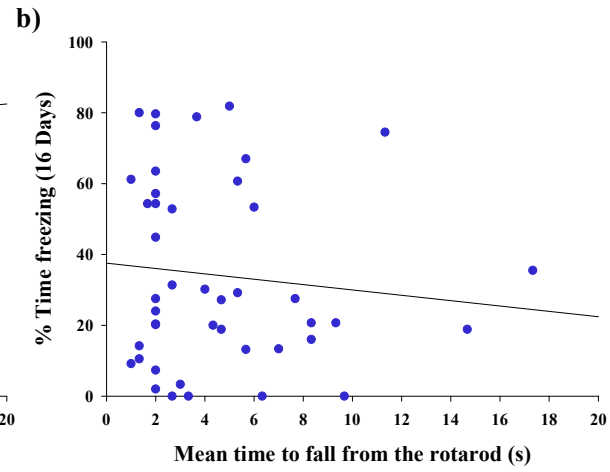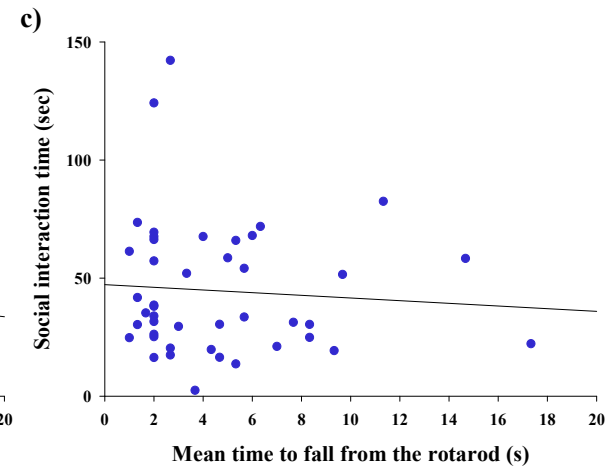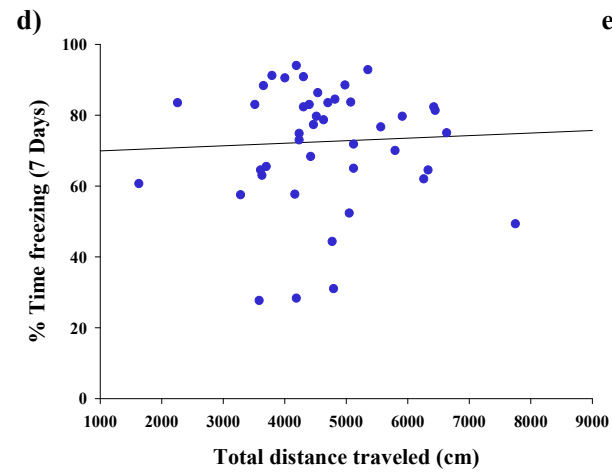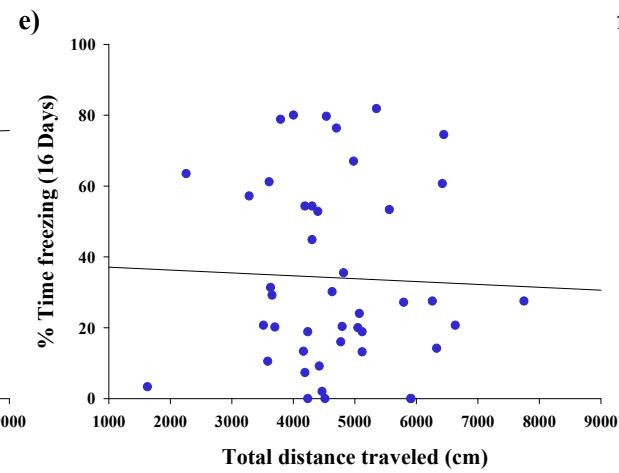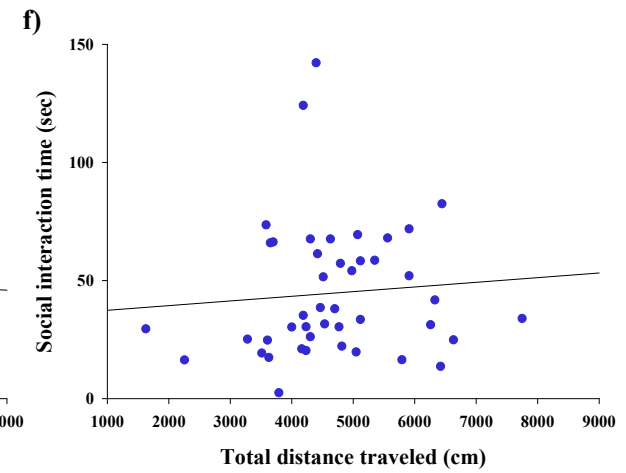

Supplement: Supplementary file 6 — Supplementary Fig. 5 [file 41398_2020_929_MOESM6_ESM.pdf]
