# Supplementary material for: Predicting susceptibility and resilience in an animal model of post-traumatic stress disorder (PTSD)
Source: Transl Psychiatry. 2020 Jul 21;10:243. doi: 10.1038/s41398-020-00929-9 (PMC7374603; doi:10.1038/s41398-020-00929-9)
Supplement: Supplementary file 7 — Supplementary Tables [file 41398_2020_929_MOESM7_ESM.docx]

| **Potential Predictive variables** | **PTSD-like alterations** | | |
| --- | --- | --- | --- |
|  | **Freezing (day 7)** | **Freezing (day 16)** | **Social Interaction Time (day 19)** |
| **Grooming frequency** | R = 0.163  P = 0.148 | R = 0.155  P = 0.170 | R = 0.023  P = 0.840 |
| **Grooming duration (s)** | R = 0.136  P = 0.230 | R = 0.191  P = 0.089 | R = 0.189  P = 0.093 |
| **Fecal boli** | R < 0.001  P = 0.998 | R = 0.032  P = 0.781 | R = 0.052  P = 0.648 |

**Supplementary Table 1. Correlation analysis for grooming, fecal boli and immobility in the Open Field test performed post trauma with the PTSD-like alterations.**

| **Potential Predictive variables** | **PTSD-like alterations** | | |
| --- | --- | --- | --- |
|  | **Freezing**  **(day 7)** | **Freezing (day 16)** | **Social Interaction Time (day 19)** |
| **Distance traveled first block**  **(0-5 min)** | R = 0.062  P = 0.643 | R = 0.082  P = 0.595 | R = 0.254  P = 0.097 |
| **Distance traveled second block**  **(5-10 min)** | R = 0.102  P = 0.510 | R = 0.034  P = 0.826 | R = 0.058  P = 0.709 |
| **Distance traveled third block**  **(10- 15 min)** | R = 0.020  P = 0.896 | R = 0.049  P = 0.755 | R = 0.037  P = 0.809 |
| **Grooming frequency** | R = 0.012  P = 0.937 | R = 0.035  P = 0.820 | R = 0.065  P = 0.673 |
| **Grooming duration (s)** | R = 0.114  P = 0.461 | R = 0.081  P = 0.601 | R = 0.094  P = 0.545 |
| **Fecal boli** | R = 0.051  P = 0.744 | R = 0.155  P = 0.314 | R = 0.149  P = 0.333 |
| **Immobility duration (s)** | R = 0.050  P = 0.745 | R = 0.085  P = 0.582 | R = 0.214  P = 0.162 |
| **Rearing frequency** | R = 0.127  P = 0.410 | R = 0.036  P = 0.816 | R = 0.174  P = 0.258 |
| **Rearing duration (s)** | R = 0.144  P = 0.350 | R = 0.068  P = 0.663 | R = 0.057  P = 0.711 |

**Supplementary Table 2. Correlation analysis for all the behavioral scores of the Open Field test performed before trauma with PTSD-like alteration.**
